# Suppression of Adiponectin by Aberrantly Glycosylated IgA1 in Glomerular Mesangial Cells In Vitro and In Vivo

**DOI:** 10.1371/journal.pone.0033965

**Published:** 2012-03-23

**Authors:** Tatsuyuki Inoue, Hitoshi Sugiyama, Masashi Kitagawa, Keiichi Takiue, Hiroshi Morinaga, Ayu Ogawa, Yoko Kikumoto, Shinji Kitamura, Yohei Maeshima, Hirofumi Makino

**Affiliations:** 1 Department of Medicine and Clinical Science, Okayama University Graduate School of Medicine, Dentistry, and Pharmaceutical Sciences, Okayama, Japan; 2 Center for iPS Cell Research and Application, Kyoto University, Kyoto, Japan; 3 Center for Chronic Kidney Disease and Peritoneal Dialysis, Okayama University Graduate School of Medicine, Dentistry, and Pharmaceutical Sciences, Okayama, Japan; Universidade de Sao Paulo, Brazil

## Abstract

The pathogenesis of IgA nephropathy (IgAN) may be associated with the mesangial deposition of aberrantly glycosylated IgA1. To identify mediators affected by aberrantly glycosylated IgA1 in cultured human mesangial cells (HMCs), we generated enzymatically modified desialylated and degalactosylated (deSial/deGal) IgA1. The state of deglycosylated IgA1 was confirmed by lectin binding to *Helix aspersa* (HAA) and *Sambucus nigra* (SNA). In the cytokine array analysis, 52 proteins were upregulated and 34 were downregulated in HMCs after stimulation with deSial/deGal IgA1. Among them, the secretion of adiponectin was suppressed in HMCs after stimulation with deSial/deGal IgA1. HMCs expressed mRNAs for adiponectin and its type 1 receptor, but not the type 2 receptor. Moreover, we revealed a downregulation of adiponectin expression in the glomeruli of renal biopsy specimens from patients with IgAN compared to those with lupus nephritis. We also demonstrated that aberrantly glycosylated IgA1 was deposited in the mesangium of patients with IgAN by dual staining of HAA and IgA. Moreover, the urinary HAA/SNA ratio of lectin binding was significantly higher in IgAN compared to other kidney diseases. Since adiponectin has anti-inflammatory effects, including the inhibition of adhesion molecules and cytokines, these data suggest that the local suppression of this adipokine by aberrantly glycosylated IgA1 could be involved in the regulation of glomerular inflammation and sclerosis in IgAN.

## Introduction

Aberrant *O*-glycosylation of IgA1 is one of the main mechanisms underlying the pathogenesis of IgA nephropathy (IgAN). Several reports have demonstrated that the aberrantly glycosylated IgA1 with reduced galactose (Gal) and/or sialic acid (Sial) and increased exposure of GalNAc was present in the sera and tonsils of patients with IgAN [1], [2], [3], [4], [5]. This suggested that desialylated and degalactosylated IgA1 (deSial/deGal IgA1) reacted with mesangial cells, which have a central role in mesangial proliferative glomerulonephritis, such as IgAN [6], and that these IgA1 altered the synthesis of mediators of inflammation which could affect cell proliferation and apoptosis [7], [8], [9] and cross-talk with other cell types, including podocytes [10].

Adiponectin, an adipocyte-derived secretory factor, promotes insulin sensitivity, decreases inflammation and promotes cell survival [11]. Adiponectin is released by adipocytes and targets a multitude of different cell types. It was first considered to be synthesized only by adipocytes; however, recent reports have indicated that it is also produced by other cell lineages [12]. The most prominent target cells are hepatocytes, cardiac myocytes [12], pancreatic beta cells, glomerular mesangial cells [13], and podocytes [14]. The two adiponectin receptors, AdipoR1 and AdipoR2, have been cloned, and these receptors appear to mediate many of the actions of adiponectin [15]. Adiponectin is an approximately 30 kDa protein that circulates in plasma as multimeric complexes at relatively high concentrations (2–10 µg/ml). Adiponectin circulates in plasma in three forms: as a trimer (low molecular weight), as a hexamer (trimer-dimer) of medium molecular weight, and as a larger multimeric high-molecular-weight (HMW) form [16], [17]. In type 1 diabetic patients, adiponectin is associated with impaired renal function [18]. There have been reports that the urinary adiponectin level is significantly associated with the serum adiponectin level and proteinuria [19] and is increased by glucocorticoid therapy in patients with IgAN [20]. However, the role of adiponectin in the regulation of glomerular inflammation has not been fully elucidated.

Direct *in situ* demonstration of aberrantly O-glycosylated IgA1 within glomerular immune deposits has recently been reported [21]. This method enabled us to perform qualitative and quantitative evaluation of aberrantly glycosylated IgA1 in routine renal biopsy samples. However, the peanut lectin binding assay utilized in the previous report was considered to be inappropriate, since this lectin could bind galactose itself. The binding characteristics of several GalNAc-specific lectins were evaluated, and lectins from Helix aspersa (HAA) and Helix pomatia bound exclusively to IgA1 containing Gal-deficient O-linked glycans [22]. Increased binding of HAA to serum IgA1 with high specificity and sensitivity has been reported in Caucasian patients with IgAN [3] and also in Japanese patients with IgAN [23], thus HAA lectin is considered to be a GalNAc-specific lectin. Therefore, we utilized *Helix aspersa* (HAA) lectin for the glomerular lectin binding assay in patients with glomerular diseases including IgAN. Moreover, both HAA and *Sambucus nigra* (SNA) lectins were applied for the urinary lectin binding assay in patients with chronic kidney diseases, and we analyzed the association between the level of lectin binding and clinicopathological findings in these patients.

Based on the literature described above, the aim of this study was to identify mediators affected by aberrantly glycosylated IgA1 in cultured human mesangial cells *in vitro*, to investigate the expression and significance of this mediator, adiponectin, in the glomeruli of patients with IgAN *in vivo*, and to develop novel lectin binding assays utilizing HAA lectins for the glomeruli of human renal biopsy specimens and utilizing HAA and SNA lectins in urine samples from patients with various chronic kidney diseases.

## Results

### Generation of desialylated and degalactosylated IgA1 (deSial/deGal IgA1) by enzymatic modification using neuraminidase and β-galactosidase

To produce deSial/deGal IgA1, human IgA was treated with neuraminidase and β-galactosidase. The efficacy of the enzymatic treatment of neuraminidase/β3-galactosidase was confirmed by a lectin-binding assay using HAA (Figure **S**1A) and SNA (Figure **S**1B), which specifically bind the terminal GalNAc and sialic acid in the hinge region of IgA1, respectively. The deSial/deGal IgA1 reacts more strongly with the HAA lectin than native IgA (OD: deSial/deGal IgA1 0.339±0.008, native IgA 0.248±0.009, *P* = 0.017), while it reacts more weakly to the SNA lectin than native IgA (OD: deSial/deGal IgA1 0.191±0.014, native IgA 0.355±0.004, *P* = 0.008).

### Adiponectin expression is downregulated and total or high molecular weight adiponectin decreases after stimulation with deSial/deGal IgA1 in cultured HMCs

**Figure 1:**
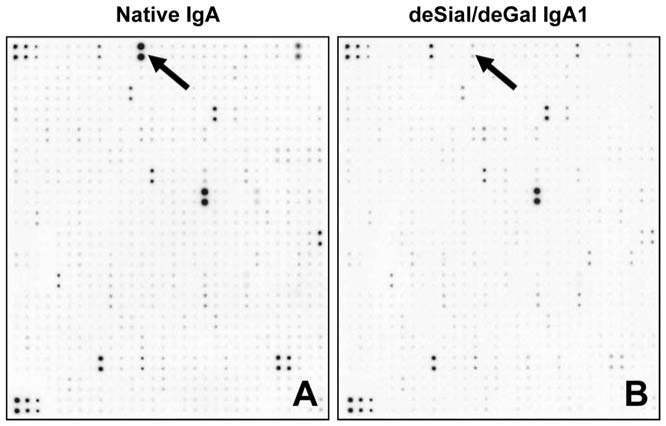
Cytokine array analysis after stimulation of cultured human mesangial cells (HMCs) with native IgA or deSial/deGal IgA1. The HMCs were stimulated with native (A) or deSial/deGal IgA1 (50 µg/ml) (B) for 48 h. The culture supernatants were then applied for a protein array analysis. After incubation of samples with array membranes for 2 h at room temperature, the spots on the membranes were scanned and digitized. The signal intensities of the spots obtained from two separate experiments were analyzed. (The proteins that were up- or downregulated by approximately 2 fold are summarized in Tables 1 and 2.) The spots shown by arrows correspond to adiponectin. The intensity of the spots in the membrane stimulated with native IgA was higher than that of cells stimulated with deSial/deGal IgA1 in HMC (A, B).

Cultured HMC were stimulated with native IgA or deSial/deGal IgA1 (50 µg/ml) for 48 h and then the culture supernatant was analyzed using a biotin label-based human cytokine array to detect the expression levels of 507 proteins (Figure 1). A total of 85 proteins were upregulated by deSial/deGal IgA1, and IL-2Rγ, angiostatin, CCR4, IL-22 BP, and thymopoietin were the five most strongly upregulated factors in the HMCs (Table **S**1). A total of 54 proteins were downregulated by deSial/deGal IgA1, and adiponectin, sFRP-3, osteocrin, TNF RI, and MMP-24 were the five most strongly downregulated factors in the HMCs (Table **S**2). The expression of TGF-β1 was also upregulated, although the expression levels of PDGF, TNF-α and MCP-1 were not. In both series of arrays of HMCs, adiponectin was dramatically downregulated (Figure 1).

**Figure 2:**
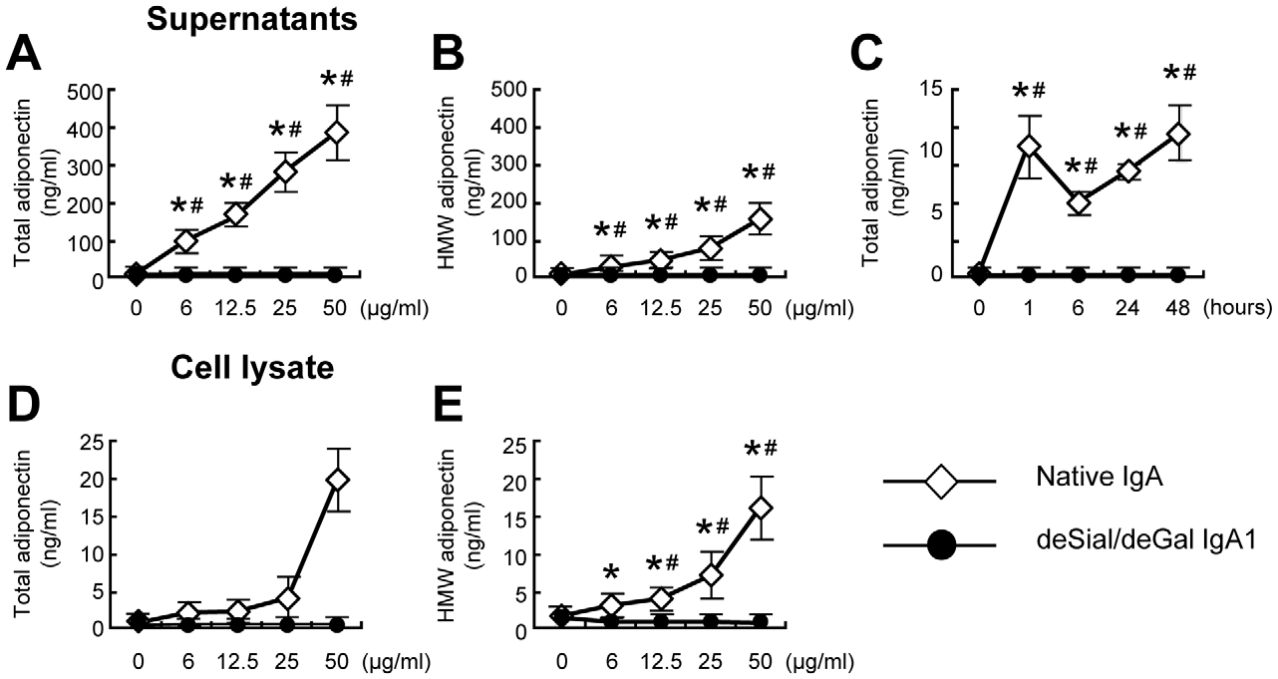
The ELISA of total (A, C, D) and HMW (B, E) adiponectin after stimulation of HMCs with native or deSial/deGal IgA1. In HMCs, native IgA upregulated the total (A) and high molecular weight (HMW) (B) adiponectin release to the supernatant in a dose- (A, B) and time-dependent manner (C). Native IgA also increased the total and HMW adiponectin concentration in cell lysates in a dose-dependent manner (D, E). HMCs were cultured with different concentrations of native or deSial/deGal IgA1 (0, 6, 12.5, 25 or 50 µg/ml) for 48 h. For the time course study, HMCs were cultured with 25 µg/ml of native or deSial/deGal IgA1 for 0, 1, 6, 24 or 48 h. The concentrations of adiponectin were expressed as the means ± SE. Open diamonds, native IgA; closed circles, deSial/deGal IgA1. *P = 0.05 vs. medium control; #*P* = 0.01, native IgA vs. deSial/deGal IgA1.

Because the impact of IgA1 on mesangial adiponectin expression is unknown, we performed a further analysis of the protein by evaluating the expression of the adiponectin protein after stimulation with native IgA or deSial/deGal IgA1 by ELISA (Figure 2). In HMCs, native IgA upregulated both the total and high molecular weight (HMW) adiponectin release to the supernatant in a dose- and time-dependent manner (Figures 2A–C). Native IgA also increased the total and HMW adiponectin concentrations in cell lysates in a dose-dependent manner (Figure 2D and 2E). Neither native IgA nor deSial/deGal IgA1 induced leptin, another adipokine, in the supernatants or cell lysates of HMCs (Figure **S**2).

**Figure 3:**
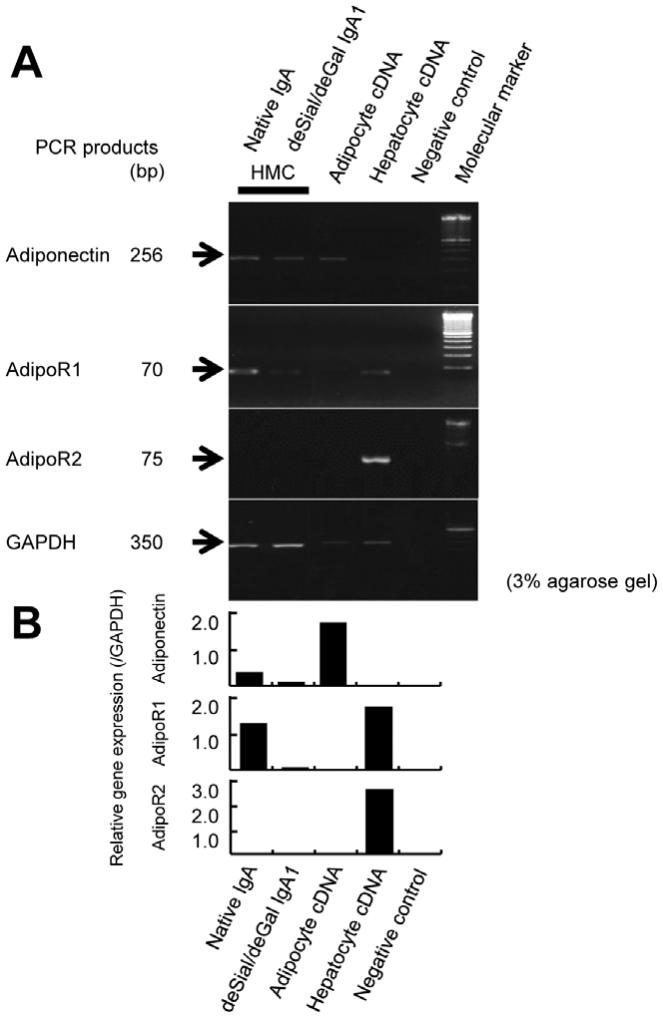
The expression of adiponectin, AdipoR1 and AdipoR2 genes in cultured human mesangial cells (HMCs). (A) The cDNA from human adipocytes was utilized as a positive control for adiponectin and the cDNA from human hepatocytes was used as a control for AdipoR1 and AdipoR2. RT-PCR for adiponectin in HMCs after stimulation with either native or deSial/deGal IgA1 afforded cDNA bands of the same size (256 bp) as that amplified from human adipocyte cDNA. RT-PCR for AdipoR1 in HMCs afforded cDNA bands of the same size (70 bp) as that amplified from human hepatocytes. The HMCs did not express AdipoR2 mRNA. The expression of the GAPDH gene was used as an internal standard (350 bp). (B) The densitometric analysis of the expression of each cDNA.

The HMCs expressed adiponectin and AdipoR1 mRNA to a lesser degree after stimulation with deSial/deGal IgA1 compared to stimulation with native IgA (Figure 3A and 3B). In contrast, the HMCs did not express AdipoR2 mRNA regardless of the form of IgA used for stimulation.

### Adiponectin expression is downregulated in the glomeruli of patients with IgA nephropathy in comparison to those with lupus nephritis or minor glomerular abnormalities

**Figure 4:**
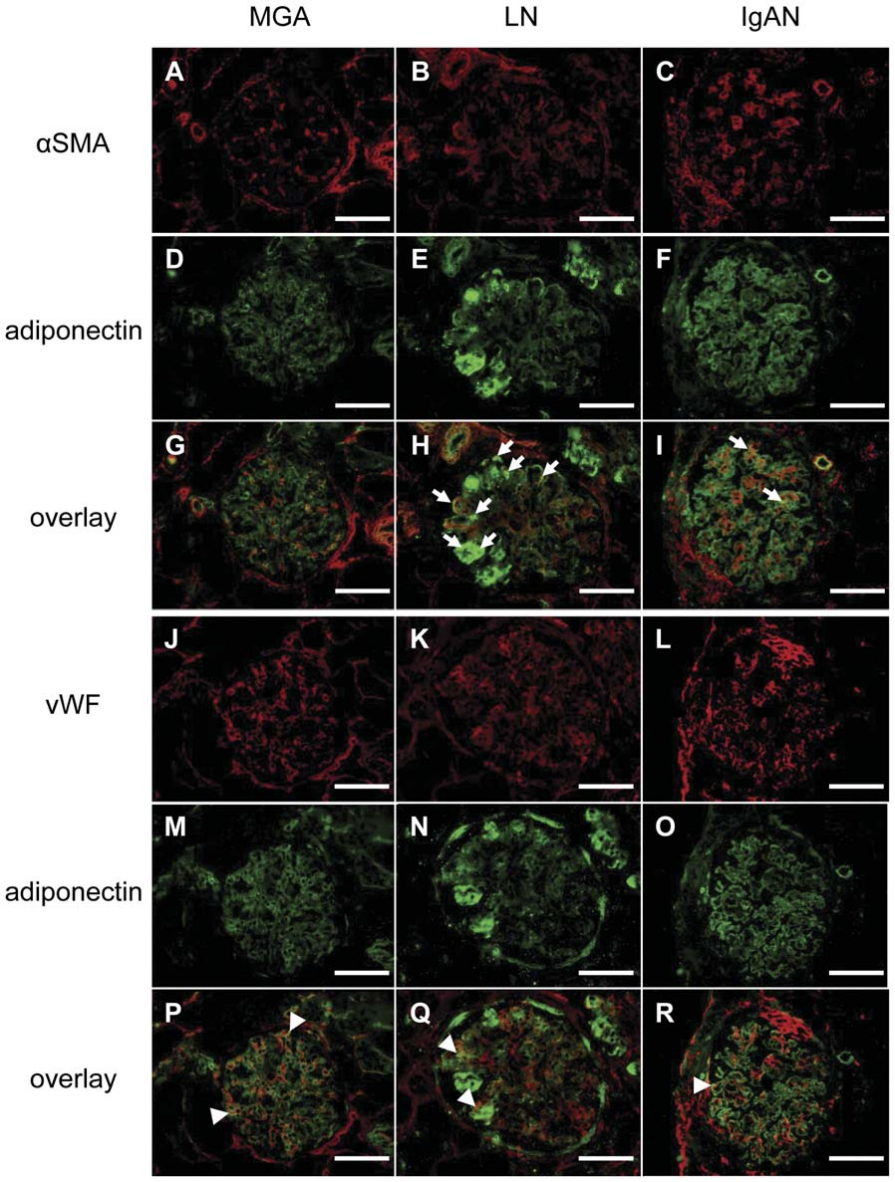
The expression of adiponectin, αSMA and vWF in human renal biopsies. Immunofluorescent staining of renal biopsy specimens from patients with minor glomerular abnormalities (MGA; A, D, G, J, M and P), lupus nephritis (LN; B, E, H, K, N and Q) and IgA nephropathy (IgAN; C, F, I, L, O and R) are shown. (Upper panels) Each section was stained for αSMA (a marker of activated mesangial cells, red) (A–C) and adiponectin (green) (D–F). Some αSMA-positive cells were colocalized with adiponectin-positive cells (yellow) (G–I). Double positive areas were predominant in the glomeruli of LN patients (H, arrows) as compared to IgAN patients (I, arrows). (Lower panels) Each section was stained for vWF (a marker of endothelial cells, red) (J–L) and adiponectin (green) (M–O). Some vWF-positive cells were colocalized with adiponectin-positive cells (yellow) (P–R, arrowheads). Double positive areas predominated in the glomeruli of MGA patients (P). Strong and segmental staining of adiponectin was recognized in the glomeruli of LN patients (E and N). The adiponectin staining in the glomeruli of IgAN patients was weaker than that of LN and MGA patients. Scale bars represent 100 µm.

We next examined the expression of adiponectin in renal biopsy specimens of patients with minor glomerular abnormalities (MGA), minimal change disease (MCD), lupus nephritis (LN) and IgA nephropathy (IgAN) by immunofluorescent staining (details in Table 1) (Figure 4). Each section was stained for αSMA (a marker of activated mesangial cells, red) (Figure 4A–C) and adiponectin (green) (Figure 4D–F). Strong and segmental staining of adiponectin was observed in the glomeruli of LN patients (Figure 4E), and some αSMA-positive cells were colocalized with adiponectin-positive cells (yellow, overlay images, Figure 4G–I, arrows). Double positive areas were predominant in the glomeruli of LN patients (Figure 4H) compared to those with MGA (Figure 4G) and IgAN (Figure 4I). Next, each section was stained for vWF (a marker of endothelial cells, red) (Figure 4J–L) and adiponectin (green) (Figure 4M–O). Some vWF-positive cells were colocalized with adiponectin-positive cells (yellow, overlay images, Figure 4P–R, arrowheads).

**Table 1:** The profiles of the patients included for analysis of renal biopsies.

	MGA (n = 9)	MCD (n = 8)	LN (n = 17)	IgAN (n = 19)
Mean age (years)	42.2±7.6	41.5±9.8	35.1±3.7	38.6±4.9
Gender (male/female)	4/5	3/5	2/15	8/11^d^
BMI (kg/m^2^)	23.3±1.0	24.6±2.1	22.5±0.9	22.1±0.8
Laboratory data				
eGFR (ml/min/1.73 m^2^)	83.0±9.1	91.0±12.9	87.9±7.7	80.9±7.2
Urinary protein (g/day)	0.7±0.5	7.0±2.0^a^	2.3±0.7^b^^, ^ c	0.9±0.7^c^^, ^ e
Hematuria (/hpf)	0.6±0.2	1.4±0.3	40.9±9.7^a^	39.9±9.2^a^^, ^ d
Serum IgA (mg/dl)	235.0±32.5	245.5±36.9	259.5±24.9	295.9±23.6
Plasma glucose (mg/dl)	97.6±4.8	125.4±22.2	101.2±7.1	101.6±6.8
HbA1c (%)	5.6±0.3	5.5±0.3	5.4±0.2	5.1±0.2
Total cholesterol (mg/dl)	208.1±24.8	355.8±48.7^b^	221.1±20.7^c^	183.6±19.5^c^^, ^ f
HDL cholesterol (mg/dl)	74.2±13.0	75.8±7.3	51.2±6.3^c^	61.1±5.7
LDL cholesterol (mg/dl)	112.4±13.1	237.5±43.1^b^	128.4±18.3^d^	97.3±15.7^d^^, ^ f
LDL/HDL ratio	1.6±0.2	3.2±0.7^b^	2.5±0.3	1.8±0.3^d^
Triglycerides (mg/dl)	126.2±22.0	202.0±28.1	182.3±20.2^b^	135.0±18.6
Pathological data				
IgA deposition (grade)	0.1±0.1	0.3±0.1	1.4±0.7^a^^, ^ c	2.3±0.1^a^^, ^ c ^, ^ e
Global sclerosis (%)	1.0±1.3	0.1±0.1	2.2±0.7	2.4±0.7
Mesangial proliferation (%)	0.0±0.0	0.0±0.0	6.9±0.8^a^^, ^ c	1.5±0.8^b^^, ^ d ^, ^ e
Mesangial sclerosis (%)	0.0±0.0	0.0±0.0	0.8±0.2	1.0±0.2^a^^, ^ c
Crescent formation (%)	0.0±0.0	0.0±0.0	2.1±0.5	1.3±0.5^b^^, ^ d ^, ^ f
Adhesion (%)	0.0±0.0	0.0±0.0	1.8±0.6	3.6±0.6^a^^, ^ c
Cell infiltration (grade)	0.2±0.2	0.1±0.1	0.9±0.2^b^^, ^ c	0.6±0.2^d^
Tubular atrophy (grade)	0.6±0.2	0.5±0.3	1.1±0.2	1.5±0.2^b^^, ^ d
Interstitial fibrosis (grade)	0.6±0.2	0.3±0.2	1.1±0.2^d^	1.6±0.2^b^^, ^ c
Vascular sclerosis (grade)	0.7±0.2	0.8±0.4	0.8±0.2	0.9±0.2

MGA, minor glomerular abnormalities; MCD, minimal change disease; LN, lupus nephritis; IgAN, IgA nephropathy; eGFR, estimated glomerular filtration rate; BMI, body mass index; HbA1c, hemoglobin A1c; HDL, high density lipoprotein; LDL, low density lipoprotein.

a : *P*<0.01,

b : *P*<0.05 vs. MGA.

c : *P*<0.01,

d : *P*<0.05 vs. MCNS.

e : *P*<0.01,

f : *P*<0.05 vs. LN.

**Figure 5:**
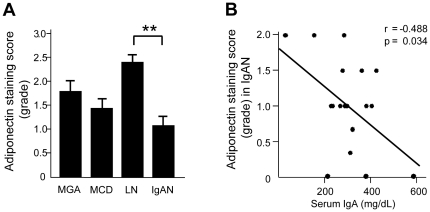
The adiponectin staining score in the glomeruli of renal biopsy specimens and the relationship between the adiponectin staining score and serum IgA in patients with IgA nephropathy (IgAN). (A) The adiponectin staining score in IgAN was significantly decreased in IgAN patients compared with that in patients with lupus nephritis (LN). Each column shown the means ± SE. MGA, *n* = 9; MCD, *n* = 8; LN, *n* = 17; and IgAN, *n* = 19. **P<0.01. (B) A significantly negative correlation was found between the adiponectin staining score and serum IgA in IgAN patients (*n* = 19) (r = −0.488, *P* = 0.034).

Semiquantitative analysis revealed that the adiponectin staining score in IgAN patients was significantly decreased compared with that in LN patients (Figure 5A). A significant negative correlation was recognized between the adiponectin staining score and the serum IgA level in IgAN patients (Figure 5B). No other clinical or pathological parameters listed in Table 1 significantly correlated with the glomerular adiponectin staining score. In vessels of renal biopsy specimens, there were no significant differences between the adiponectin expression levels among the three groups of patients (Figure S3). Using cultured human glomerular endothelial cells, we investigated the expression of total and HMW adiponectin after stimulation with native or deSial/deGal IgA1 and observed downregulation of adiponectin in these cells (Figure S4).

### Colocalization of HAA lectin with IgA deposition in the mesangium of patients with IgA nephropathy

**Figure 6:**
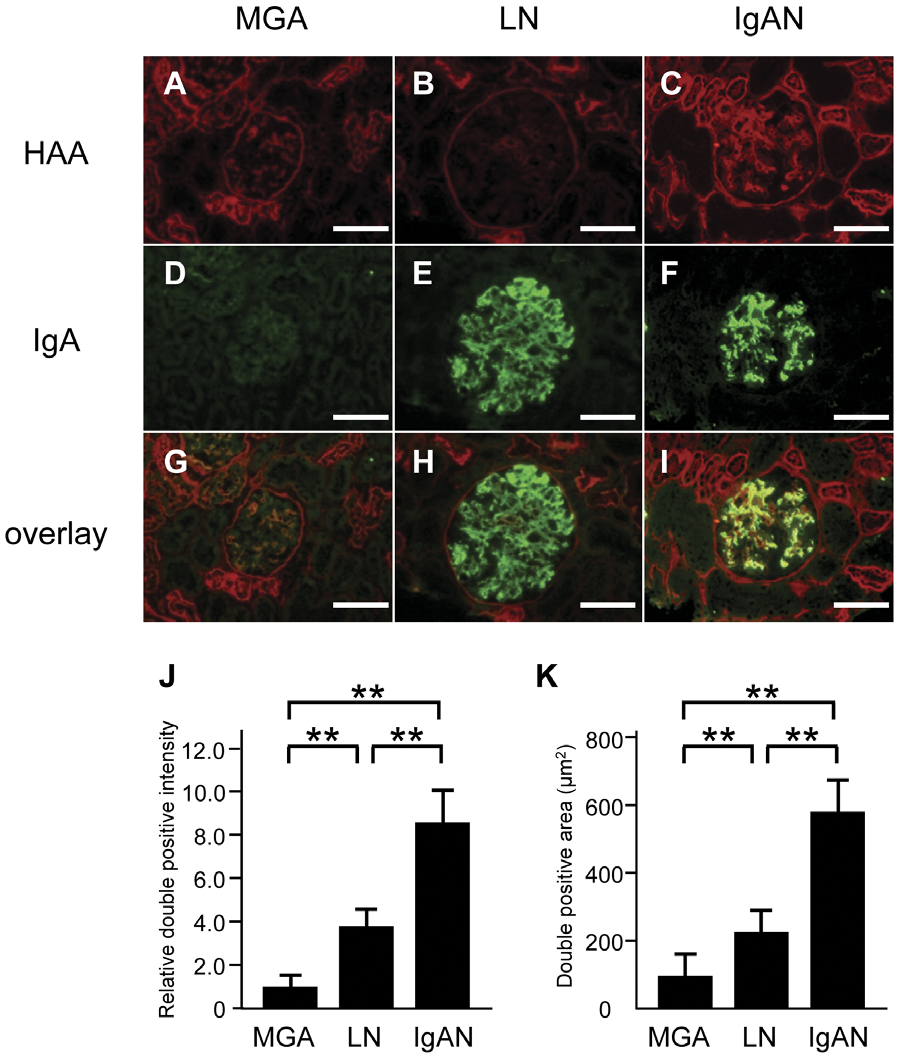
Double staining of HAA lectin and IgA in renal biopsy specimens, and the quantitative analysis of staining. Renal biopsy specimens from patients with minor glomerular abnormalities (MGA) (A, D and G), lupus nephritis (LN) (B, E and H) and IgA nephropathy (IgAN) (C, F and I) were stained for HAA lectin (red) (A–C), IgA (green) (D–F) and their images were merged (yellow) (G–I). In IgAN patients, the IgA-positive areas were colocalized with HAA lectin-positive area in the glomeruli. On the other hand, there were IgA-positive areas but no HAA lectin-positive areas in the glomeruli of LN patients. The relative double positive intensities in the glomeruli of IgAN patients were increased compared to those of LN or MGA patients (J). The double positive areas in the glomeruli of IgAN patients were increased compared to those with LN or MGA (K). Note that certain segments of tubules and Bowman's capsules were also stained with HAA lectin. Each column consists of the means ± SE. MGA, *n* = 9; LN, *n* = 14; and IgAN, *n* = 17. **P<0.01. The scale bars represent 100 µm.

We next performed double staining of HAA lectin and IgA in human renal biopsy specimens in order to clarify whether the degalactosylated IgA1 was present in the mesangium of patients with IgA nephropathy (Figure 6). Renal biopsy specimens from patients with MGA (Figure 6A, 6D and 6G), LN (Figure 6B, 6E and 6H) and IgAN (Figure 6C, 6F and 6I) were stained for HAA lectin (red) (Figure 6A–C) and IgA (green) (Figure 6D–F), and their images were merged (yellow) (Figure 6G–I). In IgAN patients, the IgA-positive areas in the glomeruli were colocalized with HAA lectin-positive areas (Figure 6I). In the glomeruli of LN patients, HAA lectin-positive areas were rarely observed and did not colocalize with IgA deposition (Figure 6H). No IgA deposition was observed in the glomeruli of MGA patients (Figure 6D). A quantitative analysis demonstrated that there was a significant increase in the relative double positive staining intensities (Figure 6J) and areas (Figure 6K) in the glomeruli of IgAN patients compared to those in LN or MGA patients. The HAA and IgA double positive areas were associated with the degree of global sclerosis of the glomeruli in IgAN patients (correlation coefficient 0.520, *P* = 0.041). No other pathological parameters listed in Table 1 significantly correlated with the HAA and IgA double positive areas.

### There is an increase in the urinary HAA/SNA ratio in patients with IgA nephropathy

**Figure 7:**
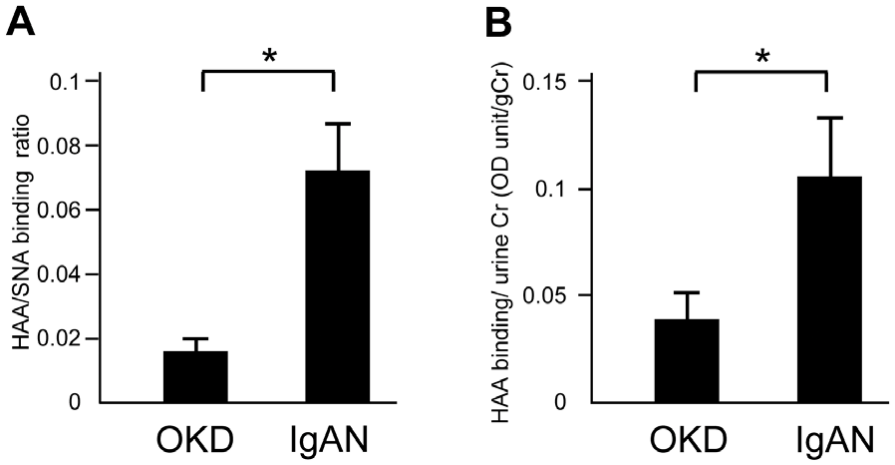
The urinary HAA/SNA binding ratio in patients with IgAN and other kidney diseases. The HAA/SNA ratios were determined by the HAA or SNA lectin binding assays using anti-IgA antibody-coated plates. The HAA/SNA ratio was higher in IgAN patients compared to patients with other kidney diseases (OKD) (*P*<0.05) (A). The level of HAA binding corrected for the urinary creatinine concentration was also higher in IgAN patients compared to patients with OKD (*P*<0.05) (B). Each column consists of the means ± SE. OKD, *n* = 142; IgAN, *n* = 78 *P<0.05.

To establish a diagnostic marker of IgAN, we determined the urinary HAA/SNA ratios using HAA and SNA lectin binding assays (Figure 7). The HAA/SNA ratio was significantly higher in patients with IgAN compared to those with other kidney diseases (OKD) including LN and MGA (*P*<0.05) (Figure 7A) (patient profiles are shown in Table 2). Moreover, the level of HAA binding corrected by the urinary creatinine concentration was significantly increased in IgAN patients compared with patients with OKD (*P*<0.05) (Figure 7B). A single regression analysis revealed there was a significant positive correlation between the urinary HAA/SNA ratio and urinary IgA in IgAN patients (correlation coefficient 0.381, *P* = 0.001). No other clinical parameters listed in Table 2 significantly correlated with the urinary HAA/SNA ratio.

**Table 2:** The profiles of the patients included in the analysis of urine samples.

	OKD(n = 142)	IgAN(n = 78)
Mean age (years)	56.9±1.4	45.3±1.8^a^
Gender (male/female)	78/75	42/36
eGFR (ml/min/1.73 m^2^)	52.2±2.5	66.8±3.1^a^
Serum IgA (mg/dl)	265.4±10.5	285.7±13.0
C3 (mg/dl)	101.4±2.3	101.1±2.5
IgA/C3	2.8±0.1	3.0±0.0
Urinary protein (g/day)	0.7±0.1	0.6±0.1
Urinary occult blood (grade)	0.4±0.0	1.0±0.1^a^
Urinary RBC (/hpf)	2.9±0.4	10.6±1.7^a^
Urinary IgA (µg/ml)	50.6±11.1	58.6±28.8
Urinary creatinine (mg/dl)	88.6±5.0	108.7±8.3^b^
HA binding (unit)	6.3±0.5	12.2±1.4
SNA binding (unit)	86.2±7.0	105.6±12.0

OKD, other kidney disease; IgAN, IgA nephropathy; eGFR, estimated glomerular filtration rate; RBC, red blood cell; HAA, *Helix aspersa*; SNA, *Sambucus nigra agglutinin*.

a : *P*<0.01,

b : *P*<0.05.

## Discussion

Our results demonstrated that adiponectin was synthesized and secreted by cultured HMCs *in vitro*. Stimulation with deSial/deGal IgA1 suppressed the secretion of adiponectin in HMCs compared to those exposed to native IgA. Moreover, we revealed that there was a downregulation of adiponectin expression in the glomeruli of renal biopsy specimens from patients with IgAN compared to those with LN or MGA. We also demonstrated that aberrantly glycosylated IgA1 was deposited in the glomerular mesangium of patients with IgA nephropathy by dual staining of HAA lectin and IgA. In addition, the urinary HAA/SNA ratio was found to be significantly higher in IgAN patients compared to those with OKD.

We observed that there was a downregulation of adiponectin expression by exposure to deSial/deGal IgA1 in HMCs and in the glomeruli of renal biopsy specimens from patients with IgAN. Initially, adiponectin was considered to be synthesized exclusively by adipocytes; however, recent studies have shown that it is also expressed by other cell types including bone-forming cells [24], endothelial cells [25], cardiomyocytes [12], bone marrow and peripheral lymphocytes [26], and airway epithelial cells [27]. The results of the present study provides the first evidence that adiponectin can be synthesized and secreted by HMCs. In addition, stimulation of HMCs with deSial/deGal IgA1 suppressed the secretion of adiponectin, but not leptin, compared to stimulation with native IgA. While it might be pointed out that the adiponectin content of mesangial cells seems to be lower than that of adipose tissues, the secretion by mesangial cells may be important, since the adiponectin secreted by mesangial cells may mediate paracrine and/or autocrine signaling pathways rather than the long-range mechanisms regulated by adipose tissues. Two receptors for adiponectin, AdipoR1 and Adipo2, have been recently cloned [15]. AdipoR1 is most highly expressed in skeletal muscle, whereas AdipoR2 is most abundantly expressed in hepatocytes. We clarified that the AdipoR1 gene, but not the AdipoR2 gene, was expressed in HMCs. A similar pattern of protein expression was previously reported in isolated rat glomeruli as determined by a Western blotting analysis [28].

Suzuki et al. demonstrated that IgA1 in the cytoplasm of IgA1-producing cell lines derived from patients with IgAN reacted with HAA lectin after treatment of the cells with neuraminidase, indicating that there was a Gal deficiency. They confirmed that the Gal-deficient IgA1 was localized to the Golgi apparatus [4]. We observed significantly more colocalization of HAA lectin with IgA deposits in the glomerular mesangium of IgAN patients compared to that of LN or MGA patients. The method we used allowed us to directly visualize the *in situ* deposition of aberrantly glycosylated IgA1 in the mesangial areas corresponding to IgA deposition; in addition, this method is easily reproducible using routine renal biopsy specimens and, importantly, is also suitable for quantitative evaluation of the relative amounts of colocalized HAA lectin with IgA molecules [21]. Moldoveanu et al. reported that a HAA-IgA ELISA could be a highly specific assay for the detection of Gal-deficiency of IgA1 *O*-linked glycan and may have potential as a non-invasive diagnostic test for IgAN [3]. Our double staining method is a novel method to distinguish IgAN from other glomerular diseases associated with IgA deposits, including LN. A recent report has indicated that another *N*-acetylgalactosamine-specific snail lectin, *Helix pomatia* agglutinin, has a comparable binding affinity for the Gal-deficient IgA1 hinge region [29] and future studies to evaluate this lectin using our glomerular lectin binding assay would be informative.

It has been demonstrated that there are aberrantly glycosylated IgA1 molecules in circulating blood, tonsils and in the mesangium of patients with IgAN [1], [2], [3], [4], [5]. In a previous study [30], we proposed that β1,3-galactosyltransferase (β3GalT) may be an important enzyme responsible for regulating the post-translational carbohydrate modification of IgA in tonsillar CD19-positive B cells in patients with IgAN, since a decrease in the expression of the β3GalT gene was significantly correlated with renal dysfunction, the degree of proteinuria, and the severity of the renal injury score. Aberrantly glycosylated IgA1 is composed of N-acetylgalactosamine (GalNAc), with or without sialic acid, and is devoid of a galactose moiety in patients with IgAN [31]. Mass spectrometry or lectin binding assays have been used for the detection of aberrantly glycosylated IgA1 in previous studies [3], [32]. The use of mass spectrometry allows detection of a difference of more detailed carbohydrate chain structures, but processing requires several complicated steps, and it was often difficult to analyze samples from IgAN patients with heterogeneous clinical states. The lectin binding assay which we utilized is convenient, with a fewer steps, and still provides specific identification and quantitation of the binding of each lectin.

We also demonstrated that the urinary HAA/SNA binding ratio and HAA binding was significantly increased in IgAN patients compared to patients with OKD. The urinary HAA/SNA binding ratio significantly correlated with the urinary IgA concentration. Galla et al. reported that monomeric IgA in the urine appeared almost exclusively in IgAN patients and patients with OKD, while polymeric IgA predominated in normal subjects [33]. With this in mind, the lectin binding assay using urine samples may be more appropriate to detect aberrantly glycosylated IgA1 than that using serum samples containing predominantly polymeric IgA. Circulating IgA may appear in the urine due to the damaged filtration properties of the glomerular capillary wall. However, we did not exclude the possibility that the lectin binding assay utilized in this study may also capture IgA-containing immune complexes.

It was previously reported that non-selective proteinuria, including high molecular weight proteins such as immunoglobulin, usually increases with the progression of kidney disease [33]. Matousovic et al. reported that immune complex deposits in the mesangium cross the filtration barrier and enter the urine because immune complex formation, including aberrantly glycosylated IgA1, may affect the isoelectoric charge [34]. The urinary HAA/SNA ratio may therefore provide a measurement of the nephritogenic form of the immunoglobulin in the urine. Several urinary biomarkers have been reported in patients with IgAN [35], [36] and recently, the urinary EGF/MCP-1 ratio was proposed as a prognostic marker [37]. It would be of interest to examine whether the urinary HAA/SNA ratio can be a prognostic marker by performing a prospective study.

This study has several limitations. First, the receptors for native or deSial/deGal IgA1 are largely unknown, so we were not able to elucidate the receptor-mediated signaling pathways that affect adiponectin expression in HMCs. There are several candidates that act as the IgA1 receptor expressed on mesangial cells. Among them, the transferrin receptor 1 (TfR1, also called CD71) has been identified [38], and the IgA1-TfR1 interaction resulted in mesangial cell proliferation [39]. Since the effects of the cellular signaling of TfR1 in mesangial cells are largely unknown, an investigation of the relationship between TfR1 and the suppression of adiponectin may be of great interest. Second, HMCs stimulated by enzymatically modified deSial/deGal IgA1, which we developed *in vitro*, may not exhibit exactly the same response as those stimulated by abberantly glycosylated IgA1 isolated from serum of human IgAN. Third, with regard to the serum adiponectin levels in patients with IgAN, Iwasa et al. reported that in males, the serum HMW adiponectin levels correlated more strongly with arteriolosclerosis of renal biopsy specimens in patients with IgAN than did the total adiponectin level [40]. We initially measured the HMW adiponectin levels in the serum from a small number of patients with IgAN and found an inverse relationship between the serum adiponectin level and the glomerular adiponectin staining score (data not shown). However, a large scale study will be needed to more precisely investigate the relationship between the serum (systemic) and glomerular (local) adiponectin levels in IgAN patients.

In conclusion, these results indicate that aberrantly glycosylated IgA1 downregulates adiponectin expression in HMCs *in vitro* and in the glomeruli of human IgAN patients. Glomerular lectin binding assays using HAA demonstrated the presence of aberrantly glycosylated IgA1 in the glomeruli predominantly in IgAN patients. The urinary HAA to SNA binding ratios may be useful diagnostic or prognostic markers for patients with IgAN. Given that adiponectin exerts anti-inflammatory effects, including the inhibition of adhesion molecules, cytokine expression and apoptosis, our data suggest that the local suppression of this adipokine by aberrantly glycosylated IgA1 could be involved in the glomerular inflammation and sclerosis in IgAN. Further studies regarding the mechanisms of downregulation of adiponectin and decreased function of the adiponectin system in the glomeruli of IgAN patients will be of particular interest.

## Materials and Methods

### Generation of deSial/deGal IgA1

To remove terminal sialic acid and expose the Gal residues, 99% purified IgA from human serum (Lot no. A01111502) (Fitzgerald Industries Laboratories, Inc., Concord, MA, USA) was incubated for 6 h at 37°C with neuraminidase (from *Streptococcus* 6646 K; Seikagaku Biobusiness Corporation, Tokyo, Japan), using 0.1 U of enzyme per mg of IgA in 0.25 M sodium acetate buffer containing 0.1% bovine serum albumin (Sigma, St Louis, MO, USA) at pH 6.5 as described [9]. A portion of the neuraminidase-treated IgA was subsequently incubated for 6 h at 37°C with β-galactosidase (from Jack beans; Seikagaku Biobusiness Corporation) to remove terminal Gal and to expose GalNAc using 0.1 U of enzyme per mg of IgA in 0.05 M citrate buffer containing 0.1% bovine serum albumin at pH 3.5 [9]. The enzyme-treated IgA1 was then dialyzed with distilled water, lyophilized, and dissolved in phosphate buffered saline (pH 7.4). (Detailed methodology is described in Methods S1.)

### Lectin binding assay

The lectin binding assay was performed as described previously with some modifications [3], [34], [41]. The 96-well microtiter plates (Corning Incorporated, NY, USA) were coated with goat anti-human IgA (SouthernBiotech, Birmingham, AL, USA) at a concentration of 1 µg/ml. The samples, including non-treated IgA (native IgA) and neuraminidase- and galactosidase-treated IgA, were added to each well, incubated overnight, and diluted to achieve comparable levels of IgA (50 µg/ml) as determined using the IgA ELISA kit (Bethyl Laboratories, Montgomery, TX, USA). Samples were then incubated with biotinylated lectins (10 µg/ml) from *Helix aspersa* (HAA) (Sigma) [3] or *Sambucus nigra agglutinin* (SNA) (Vector Laboratories, Burlingame, CA, USA) [41]. Bound lectins were measured by the addition of avidin–horseradish peroxidase conjugate (Vector Laboratories). Then, o-phenylendiamine (Sigma) was added and the absorbance was measured at 490 nm by a plate reader (Bio-Rad, Hercules, CA, USA). (Detailed methodology is described in Methods S1.)

### Culture of human mesangial cells and glomerular endothelial cells

Human mesangial cells (HMC) were purchased from Lonza (Walkersville, MD, USA), and human glomerular microendothelial cells (hGEC) were purchased from the Applied Cell Biology Research Institute (ACBRI) (Kirkland, WA, USA). The cell culture conditions were described previously [42], [43]. To examine the time course and dose-dependency of the native or deSial/deGal IgA1 on adipokine expression, the cells were cultured either with different concentrations of deSial/deGal IgA1 or native IgA for 48 h or with 25 µg/ml of deSial/deGal IgA1 and native IgA for different times. (Detailed methodology is described in Methods S1.)

### Human cytokine array

A biotin label-based human antibody array kit was purchased from RayBiotech inc. (Norcross, GA, USA). HMCs or hGECs were stimulated with native or desialylated and degalactosylated IgA1 (deSial/deGal IgA1) (50 µg/ml) for 48 h. Then, equal amounts of culture supernatants were applied to a protein array for an analysis. The membranes were developed by using an enhanced chemiluminescence-type solution, and the spots on the membranes were scanned and digitized using the Science Lab 2005 Multi Gauge software program, Ver3.0 (FUJIFILM, Tokyo, Japan). The signal intensities of the spots obtained from two separate experiments were analyzed. The intensities that changed by more than 2-fold or were decreased to less than 1/2 were considered to be upregulated or downregulated proteins after stimulation with deSial/deGal IgA1 compared to stimulation with the native IgA. (Detailed methodology is described in Methods S1.)

### ELISA

An ELISA was performed for quantification of human total adiponectin (R&D systems, Minneapolis, MN, USA), human high molecular weight adiponectin (Fujirebio Inc., Tokyo, Japan) or human leptin (R&D). (Detailed methodology is described in Methods S1.)

### RT-PCR

Total RNA was isolated from HMCs or hGECs by using an RNeasy Mini kit-based system (Qiagen, Valencia, CA) [30], [44]. The sequence of the human primer pairs and the original clones are listed in Table S3. The cDNA from human adipose tissues (Nippongene Co., LTD, Tokyo, Japan) was used as a positive control for RT-PCR of adiponectin, and the cDNA from human liver tissues (Nippongene) was used as the control for RT-PCR of adiponectin receptors. (Detailed methodology is described in Methods S1.)

### Human renal biopsy specimens and collection of urine samples

Specimens of human renal tissues were obtained by percutaneous renal biopsy. The profiles of the study patients are summarized in Table 1. The diagnosis was confirmed on the basis of clinical symptoms and immunofluorescent and light microscopic findings. In patients with LN, 9 were categorized as class IV, 5 as class III, 2 as class V and 1 was categorized as class VI. Fifteen out of 17 patients with LN were receiving corticosteroids or immunosuppressants at the time of renal biopsy. Human spot urine was collected from outpatients of Okayama University Hospital. The characteristics of these study patients are summarized in Table 2. The details of patients with other kidney disease (OKD) except IgAN are listed in Table S4. Human renal biopsy specimens and spot urine were obtained after receiving written informed consent from all patients. This study was conducted in accordance with the guidelines proposed in the Declaration of Helsinki after approval by the Institutional Review Board of Okayama University. Glomerular injury and tubulointerstitial injury were evaluated by Periodic acid-Schiff (PAS) staining and Masson trichrome staining of sections, respectively [30], [45]. (Detailed methodology is described in Methods S1.)

### Immunofluorescence

The immunofluorescence analyses were performed as described previously [30], [46], [47], [48]. The following antibodies were used as primary antibodies: goat anti-human adiponectin Ab (R&D systems), mouse anti-SMA Ab (Sigma), rabbit anti-human vWF Ab (DakoCytomation) and FITC-labeled rabbit anti-human IgA Ab (DakoCytomation) (direct immunofluorescence). In the 53 patients, adiponectin expression was graded as follows: 0, none or trace: 1, mild: 2, moderate: 3, severe. The mean score of all glomeruli in each patient was determined as the adiponectin staining score. Two independent nephrologists trained according to the training course provided by the Japanese Renal Pathology Society scored the adiponectin staining while blinded to the underlying renal diagnosis. (Detailed methodology is described in Methods S1.)

### Glomerular lectin binding assay

Double staining of HAA lectin and anti-human IgA Ab was performed to detect degalactosylated IgA1 [4], [21]. The unfixed frozen 4 µm sections of renal biopsy specimens were preincubated with an avidin/biotin blocking kit (Vector Laboratories), and stained with biotinylated HAA lectin (10 µg/ml), for 1 h at room temperature, followed by staining with Alexa-594 conjugated streptavidin or rabbit anti-human IgA Ab. We examined the double positive intensities or double positive areas in the glomeruli by a digital image analysis using the image J software program (available at http://rsb.info.nih.gpv/ij). The relative intensities in the glomeruli of IgAN or LN patients to those of MGA patients were calculated. (Detailed methodology is described in Methods S1.)

### Statistical analysis

Data, shown as the means ± SE, were analyzed by the Wilcoxon test using the JMP for windows software package version 8.0.2 (SAS Institute Inc., Cawy, NC, USA). *P* values>0.05 were considered to be statistically significant.

## Supporting Information

Figure S1 **The lectin binding assay for native IgA or deSial/deGal IgA1 to** ***Helix aspersa*** **(HAA) (A) or** ***Sambucus nigra agglutinin*** **(SNA) (B).** The efficacy of the enzymatic treatment with neuraminidase/beta3-galactosidase was confirmed by specific lectin-binding assay to HAA (A) and SNA (B), which specifically bind the terminal GalNAc and sialic acid in the hinge region of IgA1, respectively (A, B). The left two lanes of duplicate wells correspond to serial dilutions of native IgA binding to HAA (A) or SNA (B) lectin. The inset graphs show the dose response curve of the IgA concentration and HAA (A) or SNA (B) to IgA binding levels. The deSial/deGal IgA1 reacted more strongly to HAA lectin than native IgA in duplicate wells of two lanes (A, top), while it reacted more weakly to SNA lectin than native IgA (B, top). Each IgA level was adjusted to 1 µg/ml.
(TIF)

Figure S2 **ELISA of leptin after stimulation by native IgA or deSial/deGal IgA1 in human mesangial cells (HMCs) (A to C).** Neither native nor deSial/deGal IgA1 induced leptin, another adipokine, in the supernatants or cell lysates of HMCs. HMCs were cultured with different concentrations of native or deSial/deGal IgA1 (0, 6, 12.5, 25 and 50 µg/ml) for 48 h. For the time course study, HMCs were cultured with 25 µg/ml of native or deSial/deGal IgA1 for 0, 1, 6, 24 or 48 h. The concentrations of leptin were expressed as the means ± SE. Open diamonds, native IgA; closed circles, deSial/deGal IgA1.
(TIF)

Figure S3 **The expression of adiponectin, αSMA and vWF in vessels of renal biopsy specimens.** The immunofluorescent staining of renal biopsy specimens from patients with minor glomerular abnormalities (MGA; A and D), lupus nephritis (LN; B and E) and IgA nephropathy (IgAN; C and F). Merged images of αSMA (red) (a marker of vascular smooth muscle cells) and adiponectin (green) are shown in the upper panels (A to C). Merged images of vWF (red) (a marker of vascular endothelial cells) and adiponectin (green) are shown in the lower panels (D to F). Some adiponectin-positive areas were also positive for αSMA and vWF. No significant differences in the intensity and pattern of staining were observed among the three groups of patients. The scale bars represent 100 µm.
(TIF)

Figure S4 **ELISA of the total (A, C, D) and HMW (B, E) adiponectin and leptin (F to H) after stimulation with native or deSial/deGal IgA1 in human glomerular endothelial cells (hGECs).** In hGECs, native IgA upregulated the total (A) and high molecular weight (HMW) (B) adiponectin release to the supernatants in a dose- (A, B) and time-dependent manner (C). Native IgA also increased the total and HMW adiponectin concentration in cell lysates in a dose-dependent manner (D, E). Neither native nor deSial/deGal IgA1 induced leptin, another adipokine, in the supernatants or cell lysates of hGECs (F to H). The hGECs were cultured with different concentrations of native or deSial/deGal IgA1 (0, 6, 12.5, 25 or 50 µg/ml) for 48 h. For the time course study, the HMCs were cultured with 25 µg/ml of native or deSial/deGal IgA1 for 0, 1, 6, 24 or 48 h. The concentrations of adiponectin were expressed as the means ± SE. Open diamonds, native IgA; closed circles, deSial/deGal IgA1. *P = 0.05 vs. medium control; #*P* = 0.01, native IgA vs. deSial/deGal IgA1.
(TIF)

Table S1 **Upregulated proteins in the supernatants of HMCs after stimulation with deSial/deGal IgA1.**
(DOC)

Table S2 **Proteins downregulated in the supernatants of HMCs after stimulation with deSial/deGal IgA1.**
(DOC)

Table S3 **The primers used for RT-PCR.**
(DOC)

Table S4 **The details of the other kidney diseases except IgAN present in patients who provided urine samples for analysis.**
(DOC)

Methods S1 **Generation of deSial/deGal IgA1.**
(DOC)
