# Supplementary figures and images for: Suppression of Adiponectin by Aberrantly Glycosylated IgA1 in Glomerular Mesangial Cells In Vitro and In Vivo
Source: PLoS One. 2012 Mar 23;7(3):e33965. doi: 10.1371/journal.pone.0033965 (PMC3311555; doi:10.1371/journal.pone.0033965)

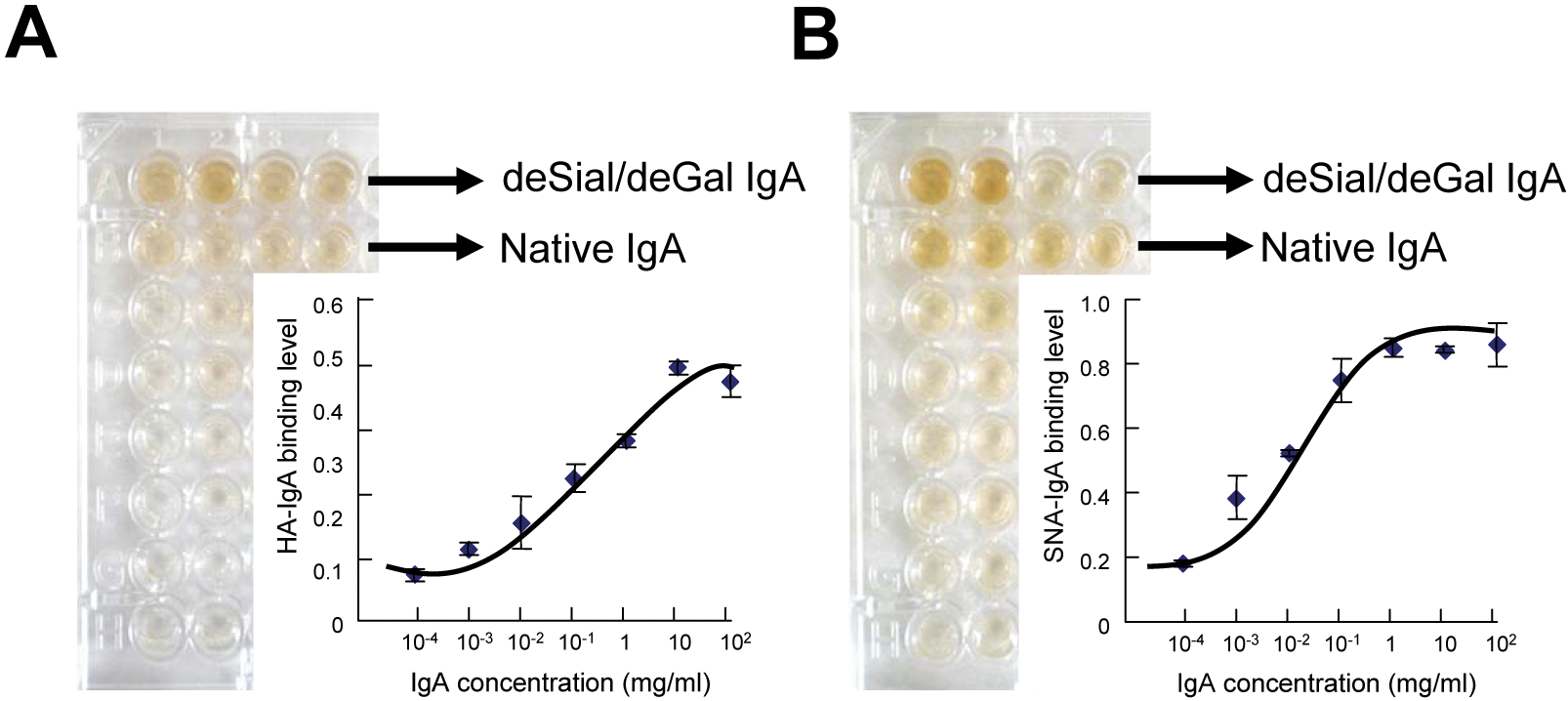

Supplement: Figure S1 — The lectin binding assay for native IgA or deSial/deGal IgA1 to Helix aspersa (HAA) (A) or Sambucus nigra agglutinin (SNA) (B). The efficacy of the enzymatic treatment with neuraminidase/beta3-galactosidase was confirmed by specific lectin-binding assay to HAA (A) and SNA (B), which specifically bind the terminal GalNAc and sialic acid in the hinge region of IgA1, respectively (A, B). The left two lanes of duplicate wells correspond to serial dilutions of native IgA binding to HAA (A) or SNA (B) lectin. The inset graphs show the dose response curve of the IgA concentration and HAA (A) or SNA (B) to IgA binding levels. The deSial/deGal IgA1 reacted more strongly to HAA lectin than native IgA in duplicate wells of two lanes (A, top), while it reacted more weakly to SNA lectin than native IgA (B, top). Each IgA level was adjusted to 1 µg/ml. (TIF) [file pone.0033965.s001.tif]

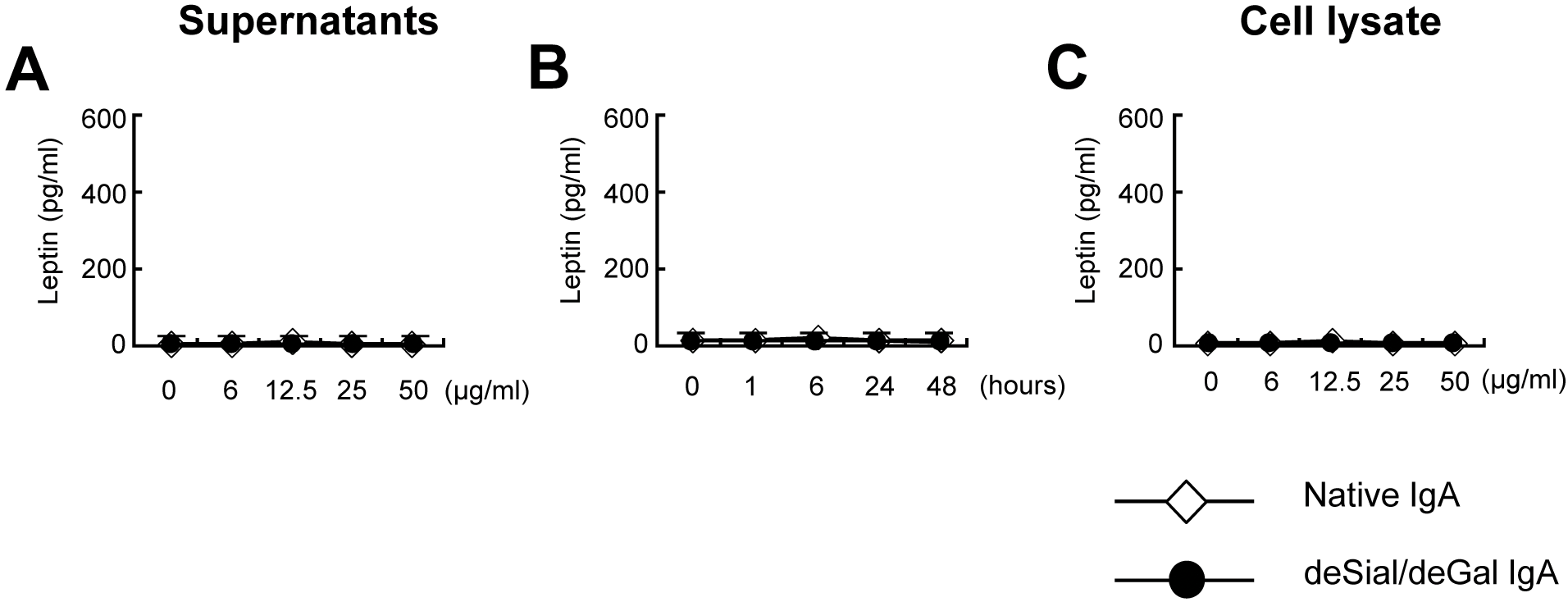

Supplement: Figure S2 — ELISA of leptin after stimulation by native IgA or deSial/deGal IgA1 in human mesangial cells (HMCs) (A to C). Neither native nor deSial/deGal IgA1 induced leptin, another adipokine, in the supernatants or cell lysates of HMCs. HMCs were cultured with different concentrations of native or deSial/deGal IgA1 (0, 6, 12.5, 25 and 50 µg/ml) for 48 h. For the time course study, HMCs were cultured with 25 µg/ml of native or deSial/deGal IgA1 for 0, 1, 6, 24 or 48 h. The concentrations of leptin were expressed as the means ± SE. Open diamonds, native IgA; closed circles, deSial/deGal IgA1. (TIF) [file pone.0033965.s002.tif]

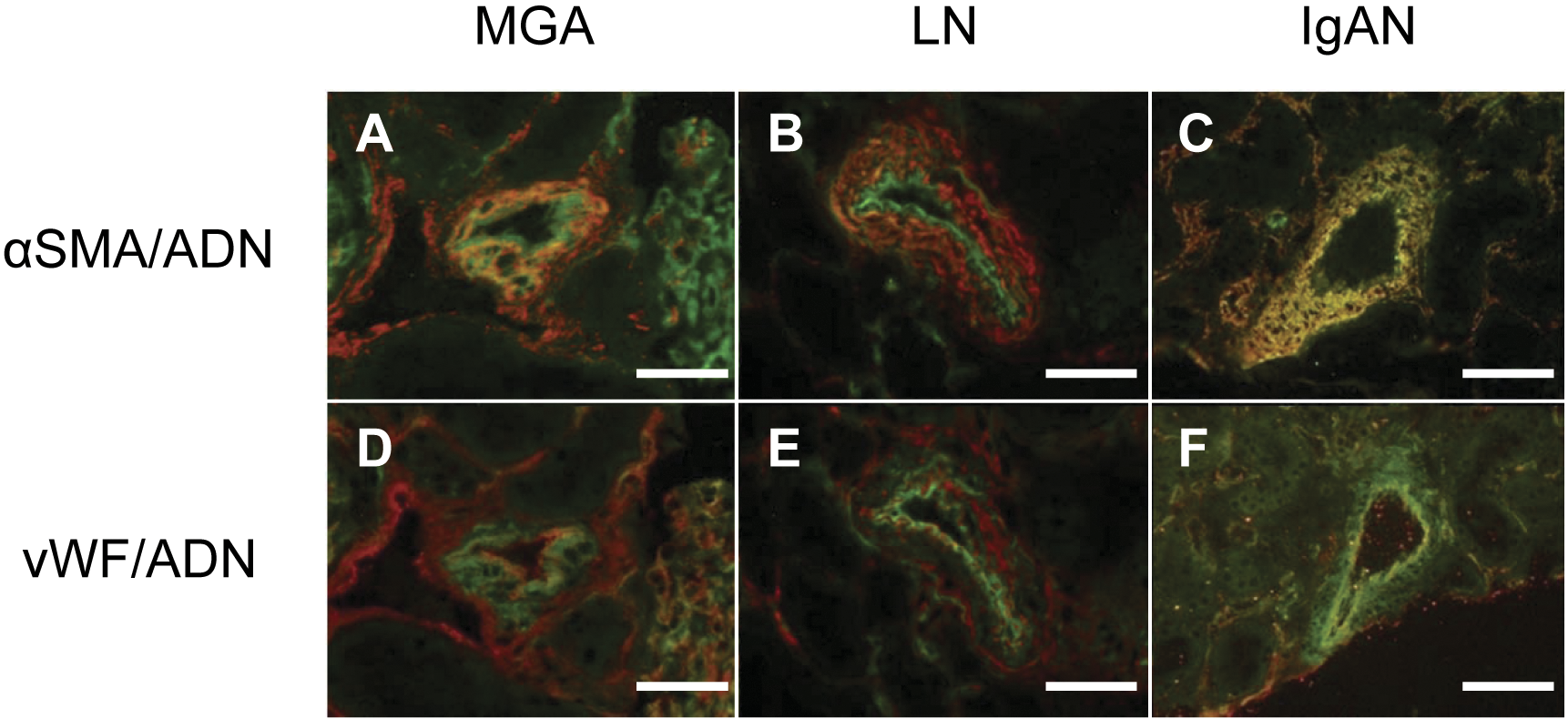

Supplement: Figure S3 — The expression of adiponectin, αSMA and vWF in vessels of renal biopsy specimens. The immunofluorescent staining of renal biopsy specimens from patients with minor glomerular abnormalities (MGA; A and D), lupus nephritis (LN; B and E) and IgA nephropathy (IgAN; C and F). Merged images of αSMA (red) (a marker of vascular smooth muscle cells) and adiponectin (green) are shown in the upper panels (A to C). Merged images of vWF (red) (a marker of vascular endothelial cells) and adiponectin (green) are shown in the lower panels (D to F). Some adiponectin-positive areas were also positive for αSMA and vWF. No significant differences in the intensity and pattern of staining were observed among the three groups of patients. The scale bars represent 100 µm. (TIF) [file pone.0033965.s003.tif]

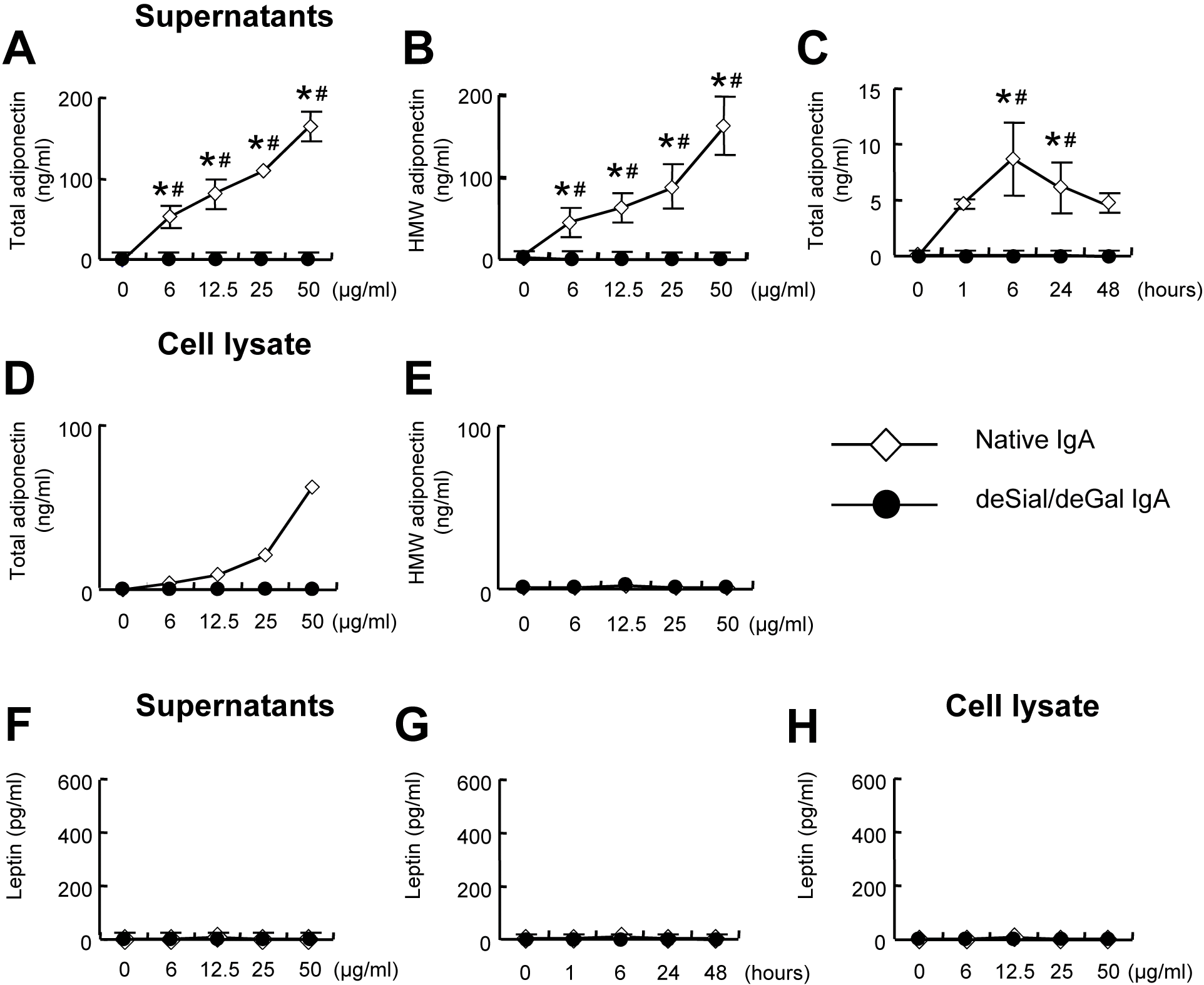

Supplement: Figure S4 — ELISA of the total (A, C, D) and HMW (B, E) adiponectin and leptin (F to H) after stimulation with native or deSial/deGal IgA1 in human glomerular endothelial cells (hGECs). In hGECs, native IgA upregulated the total (A) and high molecular weight (HMW) (B) adiponectin release to the supernatants in a dose- (A, B) and time-dependent manner (C). Native IgA also increased the total and HMW adiponectin concentration in cell lysates in a dose-dependent manner (D, E). Neither native nor deSial/deGal IgA1 induced leptin, another adipokine, in the supernatants or cell lysates of hGECs (F to H). The hGECs were cultured with different concentrations of native or deSial/deGal IgA1 (0, 6, 12.5, 25 or 50 µg/ml) for 48 h. For the time course study, the HMCs were cultured with 25 µg/ml of native or deSial/deGal IgA1 for 0, 1, 6, 24 or 48 h. The concentrations of adiponectin were expressed as the means ± SE. Open diamonds, native IgA; closed circles, deSial/deGal IgA1. *P = 0.05 vs. medium control; #P = 0.01, native IgA vs. deSial/deGal IgA1. (TIF) [file pone.0033965.s004.tif]
