# Supplementary material for: Suppression of Adiponectin by Aberrantly Glycosylated IgA1 in Glomerular Mesangial Cells In Vitro and In Vivo
Source: PLoS One. 2012 Mar 23;7(3):e33965. doi: 10.1371/journal.pone.0033965 (PMC3311555; doi:10.1371/journal.pone.0033965)
Supplement: Table S1 — Upregulated proteins in the supernatants of HMCs after stimulation with deSial/deGal IgA1. (DOC) [file pone.0033965.s005.doc]

**Table S1.** Upregulated proteins in the supernatants of HMCs after stimulation with deSial/deGal IgA1

| Protein No. | Protein expression ratio (deSial/deGal IgA1: native IgA) | Primary protein name | Protein description |
| --- | --- | --- | --- |
| 1 | 44.59553 | Interleukin-2 receptor gamma | IL-2 R gamma |
| 2 | 11.14633 | Angiostatin | Angiostatin |
| 3 | 9.88851 | CC-chemokine receptor 4 | CCR4 |
| 4 | 9.32629 | Interleukin-22 binding protein | IL-22 BP |
| 5 | 7.69277 | Thymopoietin | Thymopoietin (TP) |
| 6 | 6.25143 | CXC motif chemokine ligand 16 | CXCL16 |
| 7 | 6.03287 | Transforming growth factor-beta 1 | TGF-beta 1 |
| 8 | 6.00764 | Interleukin-17F | IL-17F |
| 9 | 5.87695 | Cytotoxic T-lymphocyte antigen-4 | CTLA-4 /CD152 |
| 10 | 5.556 | Interleukin-2 receptor alpha | IL-2 R alpha |
| 11 | 5.18559 | Connective tissue growth factor | CTGF / CCN2 |
| 12 | 4.53555 | Interleukin-22 | IL-22 |
| 13 | 4.28852 | Interleukin-13 | IL-13 |
| 14 | 4.18548 | Endocan | Endocan |
| 15 | 4.13879 | Eotaxin-3 | Eotaxin-3 / CCL26 |
| 16 | 3.98037 | Chemotactic receptor on Th2 cells | CRTH-2 |
| 17 | 3.7074 | Interleukin-2 receptor beta | IL-2 R beta (CD122) |
| 18 | 3.595 | Tumor necrosis factor ligand superfamily member 13 | APRIL |
| 19 | 3.43818 | Activin receptor type-2B | Activin RIIA/B |
| 20 | 3.41473 | Interleukin 1 family member 8 | IL-1 F8 / FIL1 eta |
