# Supplementary material for: Suppression of Adiponectin by Aberrantly Glycosylated IgA1 in Glomerular Mesangial Cells In Vitro and In Vivo
Source: PLoS One. 2012 Mar 23;7(3):e33965. doi: 10.1371/journal.pone.0033965 (PMC3311555; doi:10.1371/journal.pone.0033965)
Supplement: Table S2 — Proteins downregulated in the supernatants of HMCs after stimulation with deSial/deGal IgA1. (DOC) [file pone.0033965.s006.doc]

**Table S2.** Proteins downregulated in the supernatants of HMCs after stimulation with deSial/deGal IgA1

| こかｒles 1- new table 1iption:000000000000000000000000000000000000000000000000000000000000000000000000000000000000000000000000000Protein No. | Protein expression ratio (deSial/deGal IgA1: native IgA) | Primary protein name | Protein description |
| --- | --- | --- | --- |
| 1 | -14.3947 | Adiponectin | Adiponectin / Acrp30 |
| 2 | -8.47745 | Secreted frizzled-related protein-3 | sFRP-3 |
| 3 | -5.18753 | Osteocrin | Osteocrin |
| 4 | -4.94511 | Tumor necrosis factor receptor I | TNF RI / TNFRSF1A |
| 5 | -4.58085 | Matrix metaroprotease-24 | MMP-24 / MT5-MMP |
| 6 | -4.54669 | Matrix metaroprotease-25 | MMP-25 / MT6-MMP |
| 7 | -4.28761 | Pulmonary and activation-regulated chemokine | PARC / CCL18 |
| 8 | -4.10509 | Matrix metaroprotease-15 | MMP-15 |
| 9 | -3.8432 | Single immunoglobulin IL-1R-related molecule | SIGIRR |
| 10 | -3.73818 | Stem cell factor receptor | SCF R/CD117 |
| 11 | -3.72051 | Tyrosine-protein kinase receptor UFO | Axl |
| 12 | -3.58551 | Tumor necrosis factor receptor type 1-associated death domain | TRADD |
| 13 | -3.44341 | CC chemokine myeloid progenitor inhibitory factor 1 | MPIF-1 / CCL23 |
| 14 | -3.21099 | Tumor necrosis factor receptor superfamily member 10B | TRAIL R2 / DR5 / TNFRSF10B |
| 15 | -3.0953 | Antileukoproteinase | SLPI |
| 16 | -3.07163 | Protein NOV homolog | NOV / CCN3 |
| 17 | -2.83946 | Matrix metalloproteinase-16 | MMP-16 / MT3-MMP |
| 18 | -2.69331 | C-C motif chemokine 8 | MCP-2 |
| 19 | -2.69041 | Matrix metalloproteinase-20 | MMP-20 |
| 20 | -2.61008 | Lymphotoxin beta receptor | Lymphotoxin beta R / TNFRSF3 |
