# Supplementary material for: Suppression of Adiponectin by Aberrantly Glycosylated IgA1 in Glomerular Mesangial Cells In Vitro and In Vivo
Source: PLoS One. 2012 Mar 23;7(3):e33965. doi: 10.1371/journal.pone.0033965 (PMC3311555; doi:10.1371/journal.pone.0033965)
Supplement: Table S3 — The primers used for RT-PCR. (DOC) [file pone.0033965.s007.doc]

**Table S3**. The primers used for RT-PCR

| Protein | Primer sequences | Annealing  (°C) | Cycle No. | Product size  (bp) | GenBank  Accession # |
| --- | --- | --- | --- | --- | --- |
| Human adiponectin  Human adiponectin receptor 1  Human adiponectin receptor 2  Human GAPDH [9] | 5'- GGAGAGTGGATGATAGATGC-3'  5'- GACCTCAGTTTCTCTCAGGC-3'  5'- TTCTTCCTCATGGCTGTGATGT-3'  5'- AAGAAGCGCTCAGGAATTCG-3'  5'- ATAGGGCAGATAGGCTGGTTGA-3'  5'- GGATCCGGGCAGCATACA-3'  5'- TGAACGGGAAGCTCACTGG-3'  5'- TCCACCACCCTGTTGCTGTA-3' | 60  62  62  62 | 35  40  40  40 | 256  70  75  307 | NM_001177800  NM_015999.3  NM_024551.2  M33197 |

GAPDH, glyceraldehyde-3-phosphate dehydrogenase.
