# Supplementary material for: Suppression of Adiponectin by Aberrantly Glycosylated IgA1 in Glomerular Mesangial Cells In Vitro and In Vivo
Source: PLoS One. 2012 Mar 23;7(3):e33965. doi: 10.1371/journal.pone.0033965 (PMC3311555; doi:10.1371/journal.pone.0033965)
Supplement: Table S4 — The details of the other kidney diseases except IgAN present in patients who provided urine samples for analysis. (DOC) [file pone.0033965.s008.doc]

**Table S4.** The details of the other kidney diseases except IgAN present in patients who provided urine samples for analysis

| Disease | Number |
| --- | --- |
| Chronic glomerulonephritis  Secondary or heredity disease  Nephrosclerosis  Diabetic nephropathy  Others | 37  31  20  6  48 |
| Total | 142 |

Others include 11 cases of tubulointerstitial nephritis, 9 cases of microscopic polyangiitis, 9 cases of amyloidosis, 7 cases of gout kidney, 4 cases of malignant hypertension, 4 cases of cryoglobulinemia, 3 cases of nephrotic syndrome (minimal change nephropathy suspected) and one case of Bartter syndrome.
