# Supplementary material for: Suppression of Adiponectin by Aberrantly Glycosylated IgA1 in Glomerular Mesangial Cells In Vitro and In Vivo
Source: PLoS One. 2012 Mar 23;7(3):e33965. doi: 10.1371/journal.pone.0033965 (PMC3311555; doi:10.1371/journal.pone.0033965)
Supplement: Methods S1 — Generation of deSial/deGal IgA1. (DOC) [file pone.0033965.s009.doc]

**Methods S1**

**Generation of deSial/deGal IgA1**

To remove terminal sialic acid and expose the Gal residues, which become terminal, 99% purified IgA from human serum (Lot no. A01111502) (Fitzgerald Industries Laboratories, Inc., Concord, MA, USA) was incubated for 6 h at 37°C with neuraminidase (from *Streptococcus* 6646K; Seikagaku Biobusiness Corporation, Tokyo, Japan), using 0.1 U of enzyme per mg of IgA in 0.25 M sodium acetate buffer containing 0.1% bovine serum albumin (Sigma, St Louis, MO, USA), pH 6.5 as described with some modification . A portion of the neuraminidase-treated IgA was subsequently incubated for 6 h at 37°C with β-galactosidase (from Jack bean; Seikagaku Biobusiness Corporation) to remove terminal Gal and to expose GalNAc, which becomes terminal, using 0.1 U of enzyme per mg of IgA in 0.05 M citrate buffer containing 0.1% bovine serum albumin, pH 3.5. The enzyme-treated IgA1 was then dialyzed with distilled water, lyophilized and dissolved in phosphate buffered saline (pH 7.4).

**Lectin binding assay**

For enzyme-linked lectin-binding assays, 96-well microtiter plates (Corning Incorporated, NY, USA) were coated overnight at 4°C with goat anti-human IgA (SouthernBiotech, Birmingham, AL, USA) at a concentration of 1μg/ml diluted in a 0.05 M carbonate-bicarbonate buffer at pH 9.6. The plates were blocked with 2% bovine serum albumin in phosphate-buffered saline for 2 h at room temperature (RT). The samples, non-treated IgA (native IgA) and neuraminidase- and galactosidase-treated IgA, were added to each well and incubated overnight at 4°C. Each sample was appropriately diluted to achieve comparable levels of IgA (50 μg/ml) determined using an IgA ELISA-kit (Bethyl Laboratories, Montgomery, TX, USA). Samples were then incubated for 2 h at RT with biotinylated lectins (10 μg/ml) from *Helix aspersa* (HAA) (Sigma) or *Sambucus Nigra* *Agglutinin* (SNA) (Vector Laboratories, Burlingame, CA, USA) . Bound lectins were measured by the addition of 2.5 μg/ml avidin–horseradish peroxidase conjugate (Vector Laboratories) and incubated for 2 h at RT. After washing, 40 μg per well o-phenylendiamine (Sigma) was added and was incubated for a minimum of 20 min in the dark at RT. Finally, the reaction was terminated by the addition of 100 μl per well of 2N sulfuric acid, and absorbance was measured at 490 nm using a plate-reader (Bio-Rad, Hercules, CA, USA). The results were expressed as optical density units (OD unit).

**Culture of human mesangial cells and glomerular endothelial cells**

Human mesangial cells (HMCs) were purchased from Lonza (Walkersville, MD, USA), and human glomerular microendothelial cells (hGECs) were purchased from the Applied Cell Biology Research Institute (ACBRI) (Kirkland, WA, USA). HMCs were cultured in Dulbecco’s modified Eagle’s medium (Sigma) containing 10% fetal bovine serum (FBS). The hGECs were seeded on Attachment Factor (ACBRI)-coated cell culture flasks (Corning), and were maintained in a Cell System-Certified endothelial culture medium (ACBRI), containing the following endothelial cell growth supplements: recombinant human epidermal growth factor (10 ng/ml), bovine brain extract (12 μg/ml), hydrocortisone (1μg/ml), amphotericine B (50 ng/ml), gentamycin (50 mg/ml), and 5% (vol/vol) FBS. Growth-arrested HMCs were cultured without FBS, and hGECs were cultured with 4% FBS in a six-well plates, and incubated with deSial/deGal IgA1 or native IgA for either 4 h for RT-PCR or 48 h for ELISA. The cells were then collected for total RNA isolation and the supernatants were stored at -70°C before being analyzed by an ELISA. To examine the time course and dose-dependency of the native or deSial/deGal IgA1 on adipokine expression, the cells were cultured either with different concentrations (0, 6.2, 12.5, 25, 50 μg/ml) of deSial/deGal IgA1 or native IgA for 48 h or with 25 μg/ml of deSial/deGal IgA1 and native IgA for different times (0-48 h).

**Human cytokine array**

A biotin label-based human antibody array kit was purchased from RayBiotech Inc. (Norcross, GA, USA). Briefly, to prepare cell culture supernatants, HMCs or hGECs were plated in 100 mm tissue culture dishes. The cells were then incubated with complete culture medium at 37°C in an atmosphere of 5% CO_2_ for 48 h. The complete culture medium was replaced by media with 0% FBS for HMCs, or 4% FBS for hGECs, and then the cells were cultured for 48 h once more. The HMCs or hGECs were stimulated with native or desialylated and degalactosylated IgA1 (deSial/deGal IgA1) (50 μg/ml) for 48 h. The supernatants were collected, centrifuged at 1,000 g, aliquoted and stored at -80°C until use. Meanwhile, the cells were also collected, and the total protein concentrations were determined. For each sample, the concentration of the supernatants was determined using a total DC protein assay (Bio-Rad). Meanwhile, the membranes, which were included in a human protein cytokine array kit, were blocked with a blocking buffer. The culture supernatants were then used for the protein array analyses according to the manufacturer’s instructions. The samples were incubated with array membranes for 2 h at RT. The membranes were washed, and 1 ml of primary biotin-conjugated antibody was added and incubated at RT for 2 h. The membranes were incubated with 2 ml of horseradish peroxidase-conjugated streptavidin at RT for 30 min, then the membranes were developed by using an enhanced chemiluminescence-type solution. The spots on the membranes were scanned and digitized using the Science Lab 2005 Multi Gauge software program, Ver3.0 (FUJIFILM, Tokyo, Japan). The signal intensities of the spots obtained from two separate experiments were analyzed. The spots with intensity changes greater than 2-fold or less than 1/2 were considered to be upregulated or downregulated proteins under stimulation by deSial/deGal IgA1 compared to native IgA.

**ELISA**

An ELISA was performed for quantification of human total adiponectin (R&D systems, Minneapolis, MN, USA), human high molecular weight adiponectin (Fujirebio Inc., Tokyo, Japan) or human leptin (R&D) according to the manufacturer’s protocols. The detection sensitivities for total adiponectin, high molecular weight adiponectin, and human leptin were 0.246 ng/ml, 0.4 μg/ml and 7.8 pg/ml, while the intra-batch coefficients of variation were ± 2.8-5.0, less than ± 15, ± and 3.0-3.3 %, respectively.

**RT-PCR**

Total RNA was isolated from HMCs or hGECs by using an RNeasy Mini kit (Qiagen, Valencia, CA) as described previously . One μg of total RNA from each sample was reverse transcribed using a GeneAmp RNA PCR kit (Applied Biosystems, Foster City, CA, USA) with oligo dT primers (2.5 μmol/l), murine leukemia virus reverse transcriptase (50 U), and deoxyribonucleoside triphosphate (1 mmol/l) at 42°C for 15 min, 99°C for 5 min, and 5°C for 5 min. For the quantification of the mRNA levels of adiponectin, adiponectin receptor I (AdipoR1) and II (AdipoR2), real-time PCR was performed using the LightCycler-FastStart DNA master SYBR Green I system (Roche Diagnostic, Mannheim, Germany). Oligonucleotides for adiponectin mRNA were custom-ordered from Genostaff Co., LTD. (Tokyo, Japan), while those for AdipoR1 and AdipoR2 were from Invitrogen, and for GAPDH were from Nihon Gene Research Laboratories Inc. (Sendai, Japan). The sequence of the human primer pairs and the original clones are listed in Table S3. PCR products were analyzed by 3 % agarose gel electrophoresis and stained with ethidium bromide. Images of the gels were captured using the Printgraph software package from ATTO (Tokyo, Japan). The cDNA from human adipose tissues (Nippongene Co., LTD, Tokyo, Japan) was used as a positive control for RT-PCR of adiponectin, and the cDNA of human liver tissues (Nippongene) was used as a control for RT-PCR of the adiponectin receptors.

**Human renal biopsy specimens and collection of urine samples**

Specimens of human renal tissue were obtained by percutaneous renal biopsy. The profile of study patients is summarized in Table 1. The diagnosis was confirmed on the basis of clinical symptoms and immunofluorescent and light microscopic findings. In patients with LN, 9 were categorized in class IV, 5 were class III, 2 were class V and 1 was class VI. Fifteen out of 17 patients with LN received corticosteroids or immunosuppressants at the time of renal biopsy. Human spot urine was collected from outpatients of Okayama University Hospital. The profile of these study patients is summarized in Table 2. The details of other kidney diseases (OKD) except IgAN are listed in Table S4. Human renal biopsy specimens and spot urine were obtained after receiving written informed consent from all patients. This study was conducted in accordance with the guidelines proposed in the Declaration of Helsinki after approval by the Institutional Review Board of Okayama University. Glomerular injury was evaluated in Periodic acid-Schiff (PAS)-stained sections. The ratio of lesions (global sclerosis, mesangial proliferation, mesangial sclerosis, crescent formation and adhesion) per total number of glomeruli was scored . The observers scored the samples using a semi-quantitative scale designed to evaluate the degree of tubulointerstitial injury, including cell infiltration, tubular atrophy, and interstitial fibrosis in Masson trichrome-stained sections. The tubulointerstitial injury score, which ranged from 0-3 was determined as follows: 0, normal kidney; 1, mild changes; 2, moderate changes; 3, severe changes .

**Immunofluorescence**

Immunofluorescence was performed as described previously . The following antibodies were used as primary antibodies: goat anti-human adiponectin Ab (R&D systems), mouse anti-SMA Ab (Sigma), rabbit anti-human vWF Ab (DakoCytomation) and FITC-labeled rabbit anti-human IgA Ab (DakoCytomation) (direct immunofluorescence). In 53 patients, adiponectin expression was graded as follows: 0, none or trace: 1, mild: 2, moderate: 3, severe. The mean score of all glomeruli in each patient was determined as the adiponectin staining score. Immunofluorescence was examined under a Keyence immunofluorescence microscope (BZ-analyzer; Keyence, Osaka, Japan). The two independent investigators of the nephrologist trained by the training course of Japanese Renal Pathology Society scored the adiponectin staining blinded the underlying renal diagnosis.

**Glomerular lectin binding assay**

Double staining with the HAA lectin and anti-human IgA Ab was performed to detect degalactosylated IgA1 . The unfixed frozen 4 μm sections of renal biopsy specimens were preincubated with avidin/biotin blocking kit (Vector Laboratories), and were stained with biotinylated HAA lectin (10 μg/ml) for 1 h at room temperature, followed by incubation with Alexa-594 conjugated streptavidin or rabbit anti-human IgA Ab. We examined the double positive intensities or double positive areas in glomeruli by digital image analysis using the image J software package (available at <http://rsb.info.nih.gpv/ij>) . The relative intensities of IgAN or LN to those of MGA in the glomeruli were calculated.

**Statistical analysis**

Data, shown as the means ± SE, were analyzed by the Wilcoxon test using the JMP for windows version 8.0.2 software package (SAS Institute Inc., Cawy, NC, USA). *P* values > 0.05 were considered to be statistically significant.

**References**

1. Peruzzi L, Amore A, Cirina P, Trusolino L, Basso G, et al. (2000) Integrin expression and IgA nephropathy: in vitro modulation by IgA with altered glycosylation and macromolecular IgA. Kidney Int 58: 2331-2340.

2. Matousovic K, Novak J, Yanagihara T, Tomana M, Moldoveanu Z, et al. (2006) IgA-containing immune complexes in the urine of IgA nephropathy patients. Nephrol Dial Transplant 21: 2478-2484.

3. Moldoveanu Z, Wyatt RJ, Lee JY, Tomana M, Julian BA, et al. (2007) Patients with IgA nephropathy have increased serum galactose-deficient IgA1 levels. Kidney Int 71: 1148-1154.

4. Ding JX, Xu LX, Lv JC, Zhao MH, Zhang H, et al. (2007) Aberrant sialylation of serum IgA1 was associated with prognosis of patients with IgA nephropathy. Clin Immunol 125: 268-274.

5. Inoue T, Sugiyama H, Hiki Y, Takiue K, Morinaga H, et al. Differential expression of glycogenes in tonsillar B lymphocytes in association with proteinuria and renal dysfunction in IgA nephropathy. Clin Immunol 136: 447-455.

6. Giannakakis K, Feriozzi S, Perez M, Faraggiana T, Muda AO (2007) Aberrantly glycosylated IgA1 in glomerular immune deposits of IgA nephropathy. J Am Soc Nephrol 18: 3139-3146.

7. Suzuki H, Moldoveanu Z, Hall S, Brown R, Vu HL, et al. (2008) IgA1-secreting cell lines from patients with IgA nephropathy produce aberrantly glycosylated IgA1. J Clin Invest 118: 629-639.

8. Pineiro R, Iglesias MJ, Gallego R, Raghay K, Eiras S, et al. (2005) Adiponectin is synthesized and secreted by human and murine cardiomyocytes. FEBS Lett 579: 5163-5169.

9. Tokunaga K, Nakamura Y, Sakata K, Fujimori K, Ohkubo M, et al. (1987) Enhanced expression of a glyceraldehyde-3-phosphate dehydrogenase gene in human lung cancers. Cancer Res 47: 5616-5619.
